# The Role of Subinhibitory Concentrations of Daptomycin and Tigecycline in Modulating Virulence in *Staphylococcus aureus*

**DOI:** 10.3390/antibiotics10010039

**Published:** 2021-01-03

**Authors:** Salman Sahab Atshan, Rukman Awang Hamat, Marco J. L. Coolen, Gary Dykes, Zamberi Sekawi, Benjamin J. Mullins, Leslie Thian Lung Than, Salwa A. Abduljaleel, Anthony Kicic

**Affiliations:** 1 Department of Medical Science, Faculty of Dentistry, Basrah University, Basrah 61004, Iraq; 2 Department of Medical Microbiology, Faculty of Medicine and Health Sciences, Universiti Putra Malaysia, Serdang 43400, Selangor, Malaysia; zamberi@upm.edu.my (Z.S.); leslie@upm.edu.my (L.T.L.T.); 3 School of Public Health, Curtin University, Perth, WA 6152, Australia; garydykes66@gmail.com (G.D.); b.mullins@curtin.edu.au (B.J.M.); anthony.kicic@telethonkids.org.au (A.K.); 4 Wal-yan Respiratory Research Centre, Telethon Kids Institute, The University of Western Australia, Perth, WA 6009, Australia; 5 School of Earth and Planetary Sciences, Curtin University, Perth, WA 6152, Australia; marco.coolen@curtin.edu.au; 6 Department of Biology, Faculty of Science, Basrah University, Basrah 61004, Iraq; sal_bio2009@yahoo.com; 7 Department of Respiratory and Sleep Medicine, Perth Children’s Hospital, Nedlands, WA 6009, Australia; 8 Centre for Cell Therapy and Regenerative Medicine, School of Medicine and Pharmacology, The University of Western Australia, Nedlands, WA 6009, Australia

**Keywords:** *S. aureus*, adhesion genes, exoproteins, qRT-PCR, 2D gel SDS-PAGE

## Abstract

*Staphylococcus aureus* (*S. aureus*) infections are notoriously complicated by the ability of the organism to grow in biofilms and are difficult to eradicate with antimicrobial therapy. The purpose of the current study was to clarify the influence of sub-inhibitory concentrations (sub-MICs) of daptomycin and tigecycline antibiotics on biofilm adhesion factors and exoproteins expressions by *S. aureus* clinical isolates. Six clinical isolates representing positive biofilm *S. aureus* clones (3 methicillin-sensitive *S. aureus* (MSSA) and 3 methicillin-resistant *S. aureus* (MRSA)) were grown with sub-MICs (0.5 MIC) of two antibiotics (daptomycin and tigecycline) for 12 h of incubation. RNA extracted from culture pellets was used via relative quantitative real-time-PCR (qRT-PCR) to determine expression of specific adhesion (*fnbA*, *fnbB*, *clfA*, *clfB*, *fib*, *ebps*, *cna*, *eno*) and biofilm (*icaADBC*) genes. To examine the effect of sub-MIC of these antibiotics on the expression of extracellular proteins, samples from the culture supernatants of six isolates were collected after 12 h of treatment with or without tigecycline in order to profile protein production via 2D gel sodium dodecyl sulfate-polyacrylamide gel electrophoresis (2D gel-SDS-PAGE). Sub-MIC treatment of all clinical MRSA and MSSA strains with daptomycin or tigecycline dramatically induced or suppressed *fnbA*, *fnbB*, *clfA*, *clfB*, *fib*, *ebps*, *cna*, *eno*, and *icaADBC* gene expression. Furthermore, sub-MIC use of tigecycline significantly reduced the total number of separated protein spots across all the isolates, as well as decreasing production of certain individual proteins. Collectively, this study showed very different responses in terms of both gene expression and protein secretion across the various isolates. In addition, our results suggest that sub-MIC usage of daptomycin and tigecycline could signal virulence induction by *S. aureus* via the regulation of biofilm adhesion factor genes and exoproteins. If translating findings to the clinical treatment of *S. aureus*, the therapeutic regimen should be adapted depending on antibiotic, the virulence factor and strain type.

## 1. Introduction

*Staphylococcus aureus* (*S. aureus*) is a major problem in many clinical situations, and antibiotic-resistant forms are classified as a “high priority” pathogen by the World Health Organization (WHO) [1]. Additionally, most *S. aureus* infections are thought to involve biofilm-forming isolates [2]. Since adherence is the initial step of many infectious processes [3], the ability of antibiotics to affect this property may be an important criterion in selecting an antibiotic for clinical therapy [4]. Current strategies for treating *S. aureus* infections reduce its virulence rather than directly killing it [5]. However, whether sub-inhibitory antibiotics can effectively reduce the rate of *S. aureus* resistance is still controversial [6]. For example, in vitro data has shown that *S. aureus* α-toxin production is significantly decreased by sub-inhibitory concentrations of amoxicillin, gentamicin, and moxifloxacin [7], whereas sub-inhibitory concentrations of β-lactam antibiotics have been shown to lead to an increase in α-toxin production [8]. Linezolid has also been shown to suppress several virulence proteins [9]. A similar study conducted by Stevens et al. [10] found both clindamycin and linezolid markedly suppressed translation, but not transcription, of toxin genes in *S. aureus*. Their results suggest that protein-synthesis inhibition is an important consideration when selecting antimicrobial agents to treat infections caused by toxin-producing Gram-positive pathogens and that enhanced toxin production contributes to worse outcomes.

Vancomycin is known to inhibit the biosynthesis of the bacterial cell wall and is the most ideal drug to treat *S. aureus* infections [11]. However, increased use of vancomycin has led to the development of isolates with reduced susceptibility. The effects of sub-MIC vancomycin on adhesion factors and biofilm genes have been tested [12] as has daptomycin and tigecycline, which have been shown to be active against *S. aureus* when in biofilm formation [13]. Daptomycin is a cyclic lipopeptide antibiotic with potent bactericidal activity whose destruction of the cell membrane makes it an ideal antibiotic against most Gram-positive pathogens including vancomycin-resistant enterococci and MRSA [14]. It consists of a 13-member amino acid cyclic lipopeptide with a decanoyl side chain, which is inserted into the bacterial cell membrane, causing rapid membrane depolarization and potassium ion efflux [15]. Tigecycline is a new class of broad-spectrum antimicrobial agents known as a bacteriostatic glycylcyclines, which inhibit protein synthesis in both Gram-positive and Gram-negative organisms [16]. Using these two agents as sentinel examples of cell membrane and protein synthesis inhibitors, in the current study, we tested the in vitro effects of sub-MIC daptomycin and tigecycline on selected microbial surface components recognizing adhesive matrix molecules (MSCRAMMs) and biofilm formation, using RT-qPCR. Furthermore, the effect of sub-MIC tigecycline on the secretion of extracellular proteins produced by *S. aureus* was also assessed using 2D gel-SDS-PAGE.

## 2. Materials and Methods

### 2.1. Bacterial Isolates and Preparation of Antibiotics

Six different clinical isolates of *S*. *aureus* were utilized in this study. These isolates were received in the form of stock culture from the Medical Microbiology Laboratory, Faculty of Medicine and Health Sciences, University Putra Malaysia (UPM). They were previously garnered from Kuala Lumpur General Hospital (HKL), Malaysia. The sources of the isolates were from different infection sites of clinically ill patients. These isolates were previously characterized as different clones using SCCmec typing, *spa* sequencing, and MLST [17] and were sensitive to daptomycin and tigecycline. The isolates are known for their ability to form stable biofilms and were positive for the genes, *fnbA*, *fnbB*, *clfA*, *clfB*, *fib*, *ebps*, *cna*, *eno* and *icaADBC* (Table 1) [18]. Lyophilized daptomycin and tigecycline were purchased commercially (BOIRON, Selangor, Malaysia) and both were serially diluted in Mueller Hinton broth (MHB, Difco, Detroit, MI, USA) in 12-well microtiter plates to obtain final MICs for all the isolates. Daptomycin was also supplemented with Mueller Hinton broth containing calcium at 75 mg/L (physiological ionized Ca^2+^ concentration) and magnesium at 12.5 mg/L (SMHB-PCA). Stock solutions were then kept at −20 °C until required.

### 2.2. Quantitation of Biofilms

The ability to form biofilms was investigated for all six MSSA and MRSA isolates using the safranin microtiter plate assay according to our previously described method [19].

### 2.3. MIC Determination

Daptomycin and tigecycline were serially diluted to obtain final concentrations ranging from 0.03–16 µg/mL. An equal volume of 100 µL with ~10^6^ CFU/mL final inoculum of *S. aureus* was then added to each well containing a 1 ml stock solution of each antibiotic. Culture wells were incubated at 37 °C with constant shaking for 24 h. After incubation, concentrations at 0.5 × MIC for each respective isolate was then analyzed following Clinical and Laboratory Standards Institute (CLSI) guidelines [20]. The MIC was then read as the lowest concentration that completely inhibited bacterial growth. All experiments were done in triplicate.

### 2.4. Effects of Sub-MIC Daptomycin and Tigecycline on S. aureus Growth

An initial representative standard growth curve was established for each isolate using absorbance readings at 600 nm. Here, Mueller Hinton broth (MHB) media was inoculated with an overnight culture to achieve an inoculum of 10^6^ CFU/mL (OD_600_ = 0.03), and cultures were divided into two flasks (50 mL each). Daptomycin and tigecycline were then added to one flask at the concentration determined for each antibiotic at 0.5 MIC. The second untreated flask was used as the control. Cultures were incubated at 37 °C with gentle shaking (150 rpm) and growth monitored every ~2 h for 24 h using absorbance readings. Cells in post-exponential phase were then taken for RT-qPCR and protein studies.

### 2.5. Effect of Sub-MIC Daptomycin and Tigecycline on S. aureus Adhesion and Biofilm Gene Expression.

This experiment was designed to test the effect of cell membrane and protein synthesis inhibitors on mRNA levels of adhesion and biofilm genes mentioned (Table 2). Six different *S. aureus* isolates were grown with and without 0.5 daptomycin or tigecycline for 12 h using the same steps described above to establish a growth curve. RNA was then extracted from each isolate, purified as described [19] and immediately converted to cDNA using the RevertAid^TM^first strand cDNA synthesis kit (Fermentas).

### 2.6. Primers and Their Specificities for qPCR

Gene-specific primers (Table 2) designed and synthesized in a previous study were utilized for this study. Annealing temperatures were optimized for each primer pair by the use of melting curve analysis and by post-PCR agarose gel electrophoresis for the products obtained, and all PCR products were confirmed by sequencing (Supplementary Section S1A,B) [21].

### 2.7. Quantitative Real-Time PCR and Analysis

Transcript levels of 12 genes were measured by qPCR (Eppendorf, Selangor, *Malaysia*), utilizing Power SYBR Green Master Mix (Bio-Rad, Shah Alam, *Malaysia*), following the manufacturer’s recommended protocol. Reactions were performed in triplicate using 96-well plates and the reaction volume was set at 20 µL per sample. All reactions contained 2 µL of cDNA, 10 µL of SYBR Green Master Mix, 0.5 µL of 100 µM of each primer, and 7 µL of sterile double RNase treated water. The reaction was started with an initial denaturation at 95 °C for 5 min and 40 amplification cycles of 95 °C for 30 s, 60 °C for 20 s and 72 °C for 30 s. Expression of the 12 target genes from the antibiotic treated samples were calculated relative to the untreated samples and an endogenous control (*16S rRNA*) to normalize the sample input. Transcription levels were determined using the relative standard curve method [22] and expressed as fold change. Resultant data were then analyzed using the Relative Expression Software Tool (REST) program [23]. The experiment was performed at least twice, and values were presented as the means of triplicate measured.

### 2.8. Effects of Sub-MIC Tigecycline on Secreted Proteins

In this study, we examined the effect of tigecycline on secreted proteins of six different isolates of *S. aureus*. Here, clinical isolates of *S. aureus* were treated with tigecycline (0.06–0.25 µg/mL) for 12 h or left untreated as a control. Supernatants were then collected and analyzed using 2D gel SDS-PAGE. Protein sample preparation was conducted as previously described (Atshan et al., 2015), where proteins were concentrated via tri-chloroacetic acid (TCA) precipitation. Protein concentration was then determined, where 25 µg of purified solubilized exoproteins was passively rehydrated in 125 µl rehydration buffer containing 1% DTT (Bio-Rad Laboratories, Ltd., Shah Alam, *Malaysia*) for 14 h on a 7 cm IPG strip (GE Healthcare Biosciences, Kuala Lumpur, Malaysia). Each strip was then overlaid with 2 mL of mineral oil to prevent evaporation and urea crystallization. IPG strips were placed in an isoelectric focusing instrument (PROTEAN IEF cell) and run using the three-step protocol (Table 3). Upon completion, strips were removed from the focusing unit, rinsed with ddH_2_O, and incubated with equilibration buffer I and II as recommended by the manufacturer (Bio-Rad). Crude protein mixtures were then separated using 12% acrylamide resolving gels. These were stained using silver stain plus kit (Bio-Rad Laboratories, Ltd.), and scanned using a GS-800 Mode Imager (Bio-Rad). Comparative secretomic analysis of the six clinical isolates of *S. aureus* was then conducted using the PDQuest software package (Bio-Rad).

### 2.9. Liquid Chromatography-Mass Spectrometry (LC-MS)

Protein spots that were significantly dampened during treatment with sub-MIC tigecycline were identified and carefully excised as were their control counterparts. Excised gel pieces were then kept at −80 °C until analyzed using liquid chromatography-mass spectrometry by Proteomics International Pty. Ltd. (Nedlands, Australia).

### 2.10. Statistical Analysis

Student *t*-tests (Microsoft Excel 2007) were used to determine significant differences in the relative gene expression of treated and untreated isolates determined by qPCR. However, for comparative secretomic analysis, the PDQuest advanced 8.0.1 2D gel analysis software was used to normalize gel intensities. Student’s *t*-test (95% confidence interval) were then employed to determine any significant differences in spot intensity (*p* < 0.05) between treated and untreated samples. These experiments were performed in triplicate and repeated at least twice.

## 3. Results

### 3.1. Biofilm Quantitative Assay

**Figure 1:**
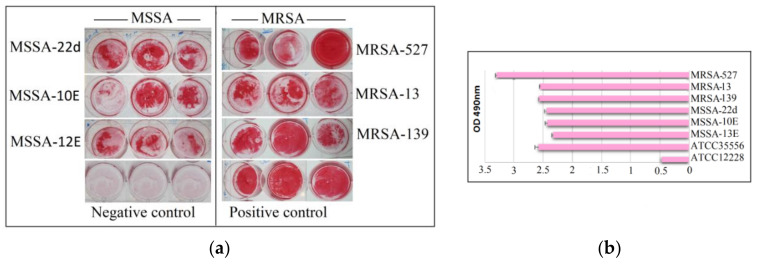
Total biofilm formation of clinical MSSA and MRSA isolates. Biofilms from all isolated were successfully formed after sufficient incubation time on microtiter plates and stained with safranin for 48 h (**a**). Semi Quantitative analysis of biofilm production measured via the optical density of destained biofilms at 490 nm (**b**). Control: indicates reference positive biofilm producer ATCC 35556 and negative biofilm producer ATCC 12228 (**a**,**b**). Columns are mean readings from triplicate wells ± SD.

Six isolates of MRSA and MSSA (Figure 1) were grown and found to be strongly adherent to inert surfaces. All isolates were found to have an OD490 values of >2.0, with MRSA-527 having the highest OD490 value, exceeding >3.0.

### 3.2. MIC Determination

All isolates assessed were found to be susceptible to daptomycin and tigecycline according to CLSI, [20] break points (Table 4). Furthermore, a very narrow range of 0.5 × MIC of both antibiotics, namely 0.06–0.25 µg/mL, was observed.

### 3.3. Effects of sub-MIC Daptomycin and Tigecyclineon Growth of S. aureus Isolates

**Figure 2:**
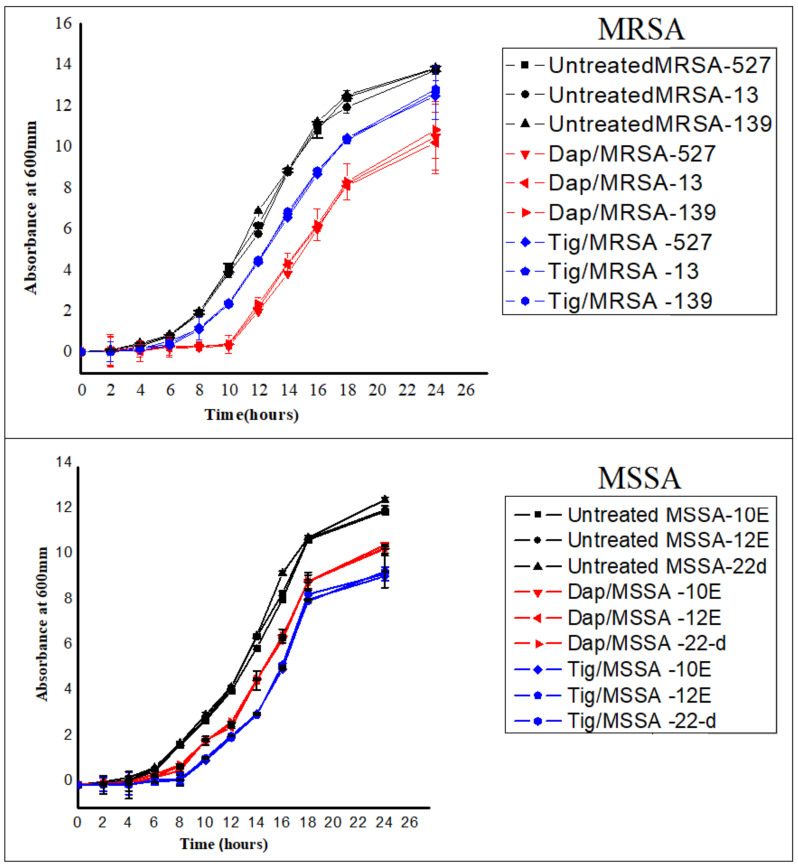
Growth curves for six different isolates of *S. aureus* [three methicillin-resistant *S. aureus* (MRSA) and three methicillin-sensitive *S. aureus* (MSSA)]. No antibiotic (untreated), 0.5 Tig (tigecycline), 0.5 Dap (daptomycin). Cultures were started with an inoculum size of 10^6^ cfu/mL and A600 nm of ~0.03, antibiotics at a concentration of 0.5 MIC were added at the early exponential phase (time zero), and cells were allowed to grow with and without antibiotics for ~24 h. Absorbances were then measured every 2 h. Values are presented as means ± SD from triplicate experiments.

As shown in Figure 2, growth curves were established for each isolate prior to and following the addition of sub-MIC levels of daptomycin and tigecycline. Untreated MRSA and MSSA isolates all actively grew over the course of the experiment (Figure 2; black curve). However, the addition of sub-MIC levels of both antibiotics resulted in slower bacterial growth, which became significant at 8 to 24 h later compared to the untreated antibiotics. Of note, 0.5 daptomycin had the best inhibitory effect on MRSA isolates (Figure 2; red curve) compared to tigecycline, while 0.5 tigecycline was more effective than daptomycin on MSSA isolates (Figure 2; blue curve).

### 3.4. Effects of Sub-MIC Daptomycin and Tigecycline Treatment on the Expression of Adhesion and Biofilm Genes

The relative expression of 12 adhesion and biofilms genes in treated isolates were calculated relative to the calibration of untreated isolates. Expression levels of adhesion and biofilm target genes were either up-regulated or down-regulated in response to the treatment among all isolates. Specifically, treatment with daptomycin induced overall target gene expression by 83.33%, 66.66%, 58.33% and 41.6% in MRSA isolate (13), MRSA (527), MSSA (10E) and MSSA (12E), respectively. Interestingly, it down regulated 83.33% of all target gene expression in MRSA (139) and 75% in MSSA (22d) (Table 5 and Supplementary Section S2A1–A6). In addition, some genes were highly up-regulated compared to other genes in the same isolate including: *fib* (22.98), *eno* (22.94), *ebps* (10.90) and *cna* (7.89) in MRSA (13), *icaA* (4.88), *fib* (2.38), *fnbB* (2.52) in MRSA (527), *can* (2.95) and *ica*C (2.57) in MSSA (10E), and *eno* (7.99), *ebps* (2.95) and *finbB* (2.79) in MSSA (12E). Sub-MIC tigecycline treatment of MSSA and MRSA was also found to modulate adhesion and biofilm gene expression in all isolates tested (Table 5 and Supplementary Section S2B1–B6). Here, tigecycline treatment caused an increase in mRNA levels in 100% of genes in MRSA (13), 83.33% of genes in MRSA (527), 58.3% of genes in MSSA (12E), 38.2% of genes in MSSA (10E). Treatment with tigecycline also caused the down regulation of 91.7% of all genes in MRSA (139) and 83.3% of genes in MSSA (22d). Minor, non-significant differences were observed with the remaining biofilm genes among the remaining isolates.

### 3.5. Effects of Sub-MIC Tigecycline Treatment on the Expression of Extracellular Proteins

**Figure 3:**
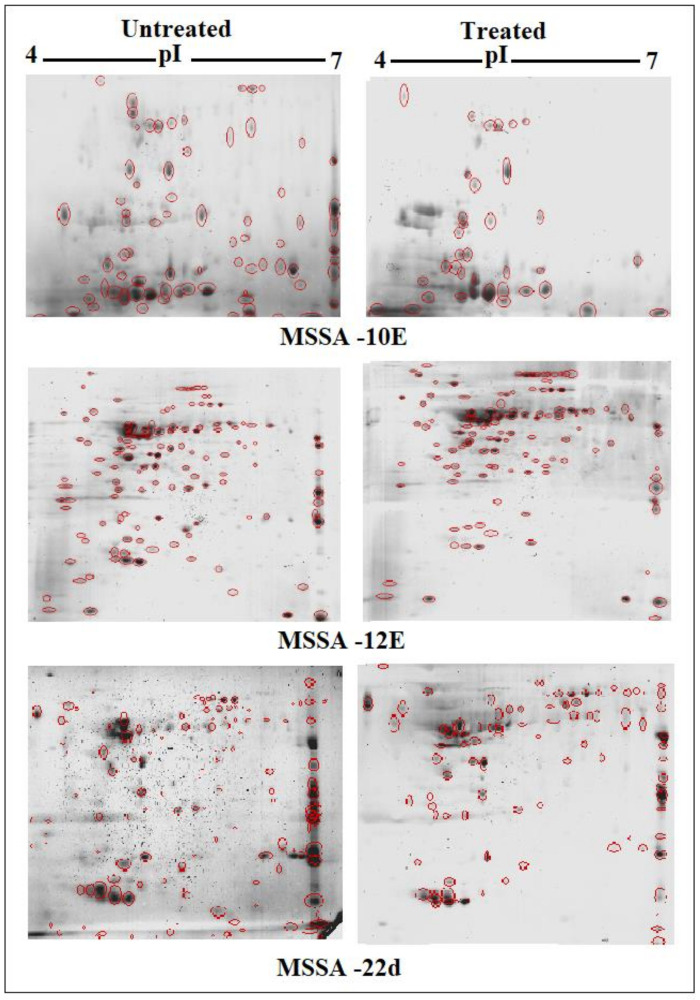
Comparison of 2D gel protein patterns of three MSSA clinical isolates with and without tigecycline treatment. In total, 25 μg of the protein extract of each isolate was separated on 2D gels, using IPG strips (pI 4–7). Protein spots were stained with silver stain and scanned using Densitometer GS-800 Mode Imager. Red circles in each gel indicate protein spots of MSSA isolates before and after treatment with 0.5 tigecycline that were separated on 2D gels. These were then calculated by using PDQuest analyses software.

**Figure 4:**
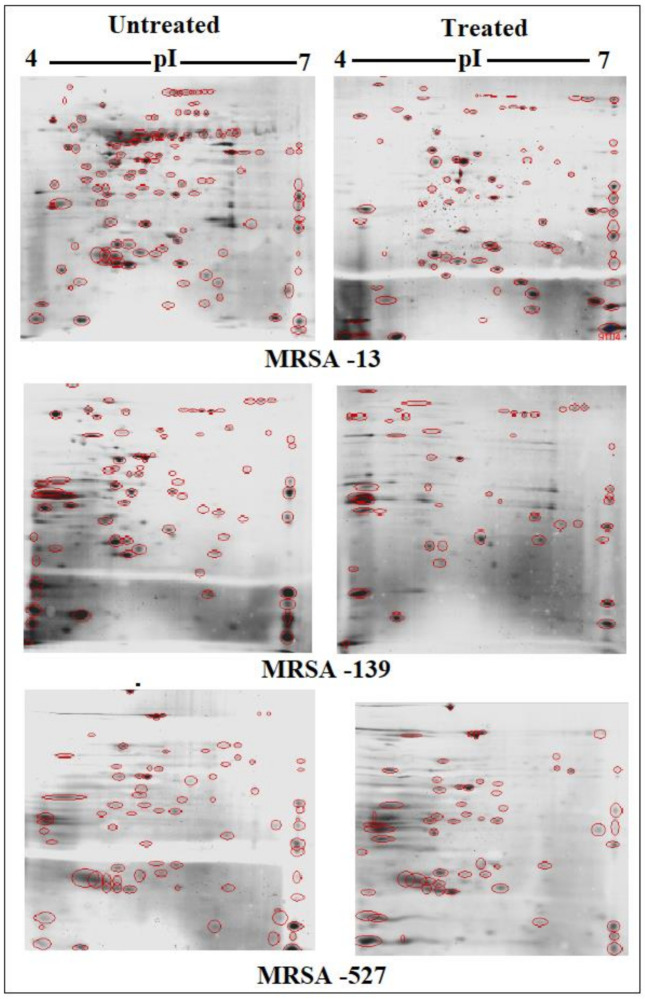
Comparison of 2DE gel protein patterns of three MRRA clinical isolates with and without tigecycline treatment. In total, 25 μg of the protein extract of each isolate was separated on 2D gels, using IPG strips (pI 4–7). Protein spots were stained with silver stain and scanned using Densitometer GS-800 Mode Imager. Red circles in each gel indicate protein spots of MRSA isolates before and after treatment with 0.5 tigecycline that were separated on 2D gels. These were then calculated by using PDQuest analyses software.

**Figure 5:**
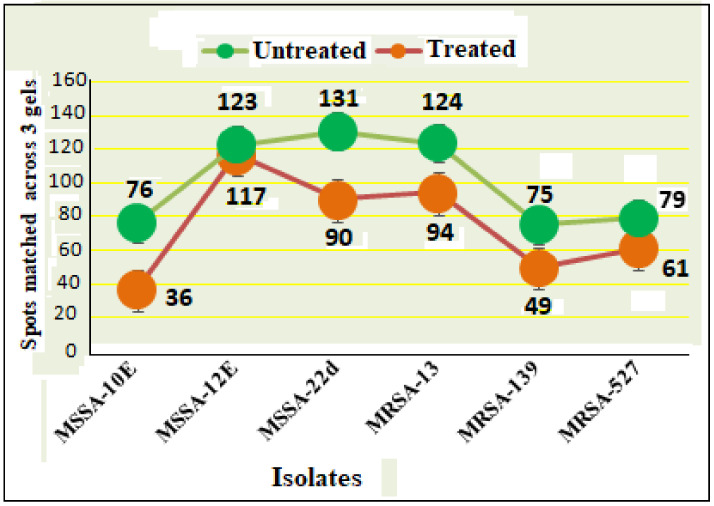
Secreted protein profiles of six clinical isolates of *S. aureus* with and without 0.5 tigecycline treatment. Numerical values on the actual graph represent number of extracellular proteins (spots) among the six isolates before (green circle) and after treatment (pink circle) with 0.5 tigecycline. Bars indicate scans from three independent experiments; data was normalized using the regression model recommended by PDQuest software (BioRad).

**Figure 6:**
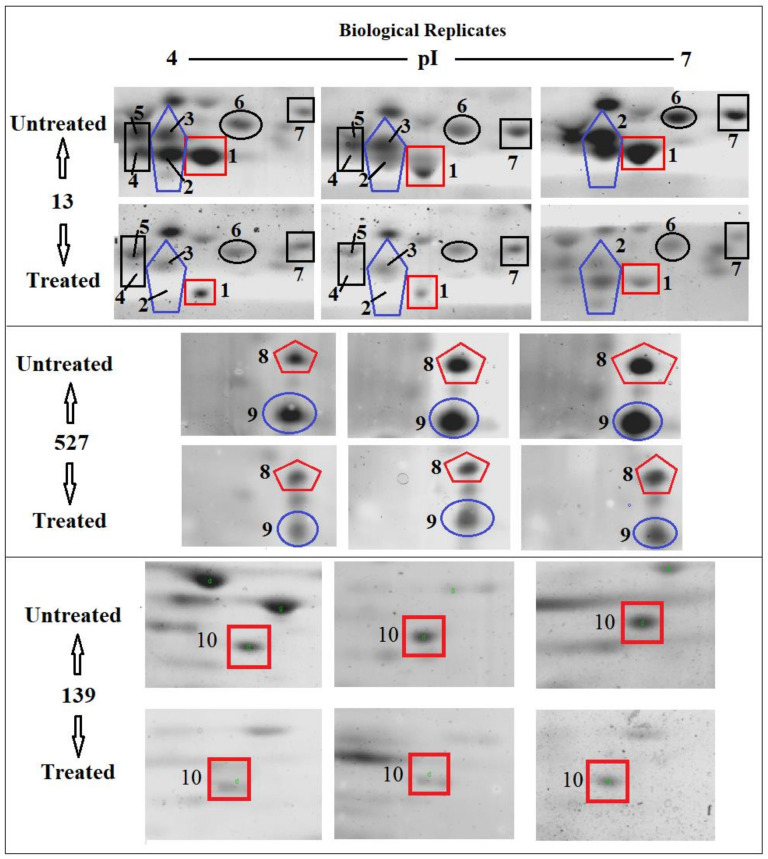
Differences in the quantities of selected protein spots in *S. aureus* clinical isolate MRSA (13), MRSA (527) and MRSA (139) with and without tigecycline treatment. The tag number (1–10) indicates a spot that was down-expressed significantly after treatment with tigecycline. These spots were selected to identify the protein by LC-MS.

Scans from three independent experiments were compared to determine differences in protein quantities between treated and untreated isolates (Figure 3 and Figure 4; Supplementary Section S3A,B). A comparison between secreted protein quantification pre- and post- treatment with tigecycline was then made (Figure 5). Secreted proteins displayed marked differences both in number as well as in spot intensity after treatment with tigecycline. For example, 40 secreted proteins were decreased in MSSA (10E) after treatment with tigecycline. Similar trends were observed in all other isolates with 6, 41, 30, 26, and 18 secreted proteins decreased in MSSA (12E), MSSA (22d), MRSA (13), MRSA (139), and MRSA (527), respectively. In addition, the intensity of each protein expressed pre- and post- treatment was also assessed. Results generated found that there was only a decrease in the number and intensity of the protein spots after treatment with tigecycline. Here, 10 strongly expressed spots were then identified via LC-MS, like Putative uncharacterized protein, Alkaline shock protein 23, Alkyl hydroperoxide reductase subunit C, protein SA21194_0967, Superoxide dismutase, Arabinose efflux permease family protein, Alcohol dehydrogenase, propanol-preferring, Exotoxin 15, Putative Cytochrome c4, and Putative septation protein spoVG (Table 6, Figure 6).

## 4. Discussion

Management of *S. aureus* infections has been hampered not only because of the increasing resistance to antibiotics but also because of the need to modulate bacterial virulence to reach clinical efficacy [24,25]. Virulence factors associated with bacterial attachment and biofilm development occur in the early exponential growth phases of infection and remain challenging to treat [26]. Prior work has evaluated virulence modification by sub-clindamycin, linezolid and beta-lactams but not for antibiotics within a similar class or with a similar mechanism of action [27]. Currently, some studies have shown that antivirulence therapy may be a potential treatment strategy in the post-antibiotic era [28]. Wang and colleagues also found that quercetin, a natural compound, can protect rats from catheter-related *S. aureus* infections by inhibiting coagulase activity [29]. In the present study, we report the modulatory effects of sub-inhibitory concentrations of daptomycin and tigecycline on *S. aureus* biofilm formation. These antibiotics were selected since they inhibit critical steps in the initial infection process, namely bacterial cell membrane adhesion and protein synthesis, which may be important considerations when selecting antimicrobial agents to combat staphylococcal infections. Initially, daptomycin and tigecycline were added to the exponential phase of bacterial growth, so that bacterial cells were active and were in the same physiological state. Growth curves were then established for each isolate used after the addition of sub-MIC levels of two antibiotics. Our results showed that daptomycin had a greater effect on MRSA isolates than tigecycline, with an opposite effect on MSSA isolates being observed. Despite this, both antibiotics did result in slower bacterial growth compared to untreated controls. These findings are similar to a previous study conducted by the same group, when using higher concentrations [13]. However, since we were targeting the start of bacterial adhesion, it is important to know whether there were any effects of daptomycin and tigecycline on adhesion factors and biofilm formation, especially since the clinical efficacy of antibiotics is not only estimated by their respective bactericidal or bacteriostatic activity, but also by their action on bacterial virulence factor release [30].

Results showed that exposure of *S. aureus* to sub-inhibitor concentrations of daptomycin significantly up-regulated the expression of the major biofilm-associated genes *MSCRAMM*, *icaA*, *icaD*, *icaB*, and *icaC*, in MRSA (13), MRSA (527) and MSSA (10E). In contrast, these genes were significantly decreased in MSSA (22d) and MRSA (139). However, most of the genes were not affected in MSSA (12E). Even with the use of the protein synthesis inhibitor, tigecycline, the effects on adhesion and biofilm genes were similarly modulated with an up-regulation of *MSCRAMM*, *icaA*, *icaD*, *icaB*, and *icaC* in most isolates. MSSA (10E) also showed that most genes were unaffected upon exposure to sub-inhibitor concentrations of tigecycline. This is a particularly important observation, since a positive gene response to these antibiotics, even at low concentrations, may imply a worse clinical outcome [31]. It is probable that daptomycin and tigecycline treatment generate signals in diverse physiological pathways, which are recognized by multiple signal sensors that in turn activate multiple response regulators including the genes measured in this study [32]. Alternatively, tigecycline and daptomycin may induce stress conditions that interact on regulatory genes, in particular genes involved in two-component regulatory systems (TCSs) such as the *agr, saeRS, srrAB, arlSR* and *lytRS*, or the recently identified *SarA* homologues (*SarR*, *Rot*, *SarS*, *SarT*, *SarU*) [33]. However, the conditions that activate TCSs are diverse and include exposure to antibiotics as well as other conditions inside the host and the resulting regulatory action often involves activation of antibiotic defenses and changes to cell physiology that in turn increases antibiotic resistance or induces cell surface modifications, and promotes biofilm formation [34]. It has also been suggested that tigecycline at sub-MIC binds to their known target sites on the ribosome, causing minor perturbations in ribosome function [27]. These effects may be responsible for a mechanism coupling transcription to translation, resulting in promoter-selective modulations of the former. The transmission of signals from ribosome to RNA polymerase due to sub-MIC of these antibiotics could also involve the release of small amounts of incomplete polypeptides, interference with ribosome assembly, induction of translation errors, or possibly interactions of small molecules with RNA [35]. Similarly, it was previously shown that sub-MIC of clindamycin stimulates synthesis of some MSCRAMM at transcription levels [36].

Due to the perplexing findings in mRNA levels observed, which may occur either by transcriptional modulation or by post transcriptional mechanisms involving mRNA turnover, an additional objective of this study was to determine whether secreted proteins expression levels correlated to mRNA expression. Using 2D SDS-PAGE, we were able to show that 608 secreted proteins were produced by the six different isolates before treatment with tigecycline in which 2DE images from the internal pooled standard were employed as a reference for comparative analyses using the PDQuest advanced Software. The number of secreted proteins (spots) were reduced to 447 after the treatment with tigecycline as shown in Figure 5. Moreover, intensity of identified spots were more than two-fold (*p* < 0.05) between treated and untreated isolates, and the rate of secreted protein production was found to decrease with tigecycline treated isolates compared to those that were untreated (Figure 6). This contrasts to findings made in another study that observed no effect on extracellular proteins in *S. aureus* strains when treated with tigecycline at sub-MIC levels [37]. One explanation for our findings is that incubation with sub-MICs of tigecycline may suppress signals in certain general metabolic pathways. One example is the two-component signaling pathway that contains a histidine kinase (HK), which responds to extracellular stimuli. Here, we show that *S. aureus* can be deprived of its complete sensorial two-component systems network and can still survive and replicate at low antibiotic concentrations [38]. In principle, protein synthesis inhibitors interact directly with the ribosome and stop peptide translation from mRNA [27]. Therefore, it is puzzling to have observed a decrease in exoprotein levels since the synthesis of negative regulators was inhibited. However, this may not have removed all suppressive effects and translation was affected as an indirect effect of antibiotic treatment. Another possibility is that the spot size of some proteins that are essential for bacteria to survive stressful conditions did not change or increase after tigecycline treatment [39].

In this experiment, some secreted proteins production was found to be highly reduced in terms of spot intensity after treatment with tigecycline (Figure 6). When some were identified by LC-MS, a number were found not to be typically exoproteins and more cytosolic in nature [40]. The presence of alkaline shock protein 23 ‘*asp23*′ in this study corroborates that of Goerke et al. [41] in which its activity would peak upon entry into the stationary growth phase when *S. aureus* are grown under stressful conditions. Work conducted previously has found higher *asp23* expression in older biofilms [42] as well as the up-regulation of σ^B^ [41]. The alternative sigma factor, σ^B^, and the accessory global regulator locus, *agr*, are two important virulence regulatory genes, which regulate the expression of several exo- and surface proteins in response to changing environmental conditions. Specifically, the effects seen with *asp23* did indeed relate to altered *sigB* transcription, this commonality is no surprise, since exoprotein repression by σ^B^ is mediated through reduced *agr* activity, and the extracellular proteases are regulated in a similar, *agr*-dependent manner [43]. However, since the sensitivity of 2D SDS-PAGE does not yield an accurate quantification of weakly expressed proteins, the overall mean coefficient of variation was high among the six isolates before and after treatment with tigecycline. Furthermore, similarities in secreted proteins were also observed and found across the various isolates, since they shared the same position (location), and number or intensity of protein spots on the gel map as a result, variations in expression of these proteins may not have been identified [44]. Moreover, variations in the mRNA levels induced by sub-MICs of antibiotics do not always result in changes in exoprotein synthesis, which should be taken into account before further conclusions are drawn [45].

## 5. Conclusions

In conclusion, different responses in terms of both gene expression and protein secretion were found across all isolates in the present study, taking into account the up- and down-modulation of the expression of MSCRAMMs, *icaA*, *icaD*, *icaB*, and *icaC* genes, may be explained by the different transcription changes that occur in the presence of each antibiotic at low concentration. Despite this, tigecycline was shown in this study to be effective at dampening both the number and quantity of secreted proteins, even when a variety of the clonal types of *S. aureus* were used. Findings suggest that the ability of these antibiotics to efficiently treat clinical *S. aureus* infections may vary between strains. Further studies are needed to test if these antibiotics also act on the release of adhesion and biofilm genes in vivo, especially when present at suboptimal concentrations, and whether this action proves to be beneficial for patients with strain-specific *S. aureus* infections.

## Figures and Tables

**Table 1:** The *S. aureus* isolates used in this study.

Strain/Ho.Des.No.	*spa* Types	MLST	SCCmec	* Adhesion and Biofilm Genes	* Antibiotic Susceptibility	Isolation Site
		ST CC				
MRSA-527	t037	ST-239 CC8	IIIA	+	S	Pus swab
MRSA-13	t4150	ST-239 CC8	IIIA	+	S	Wound swab
MRSA-139	t138	ST-1283 CC8	IIIA	+	S	Blood
MSSA-10E	t084	ST-15 CC15	V	+	S	CSF
MSSA-12E	t701	ST-152 CC8	V	+	S	Hematoma
MSSA-22d	t548	ST-5 CC5	V	+	S	Urine

Ho.Des.No. (hospital designation number), MRSA (methicillin resistant *Staphylococcus aureus*), MSSA (methicillin sensitive *Staphylococcus aureus*), *spa* (Staphylococcal surface protein A typing), MLST (multi locus sequence typing with their clonal complex(cc), SCCMec (Staphylococcal Cassette Chromosome mec *typing*), + (positive), * adherence genes (*fnbA*, *fnbB*, *clfA*, *clfB*, *fib*, *ebps*, *cna*, *eno*), and biofilm genes (*icaADBC*), * Antibiotic (daptomycin and tigecycline), S (sensitive), *CSF* (cerebrospinal fluid).

**Table 2:** Sequences of oligonucleotide primers used for qPCR.

Genes	Nucleotide Sequence of Primers (5′-3′)	Accession Numbers	Annealing Temperature	Amplicon Size (bp)
*ica*A	5-GAGGTAAAGCCAACGCACTC-3	AF086783	* 60	151
	5-CCTGTAACCGCACCAAGTTT-3			
*icaD*	5-ACCCAACGCTAAAATCATCG-3	AF086783	60	211
	5-GCGAAAATGCCCATAGTTTC-3			
*icaB*	5-ATACCGGCGACTGGGTTTAT-3	AF086783	60	140
	5-T TGCAAATCGTGGGTATGTGT-3			
*icaC*	5-CTTGGGTATTTGCACGCATT-3	AF086783	60	209
	5-GCAATATCATGCCGACACCT-3			
*fnbA*	5-AAATTGGGAGCAGCATCAGT-3	X95848.1	60	121
	5-GCAGCTGAATTCCCATTTTC-3			
*fnbB*	5-ACGCTCAAGGCGACGGCAAAG-3	X62992.1	60	197
	5-ACCTTCTGCATGACCTTCTGCACCT-3			
*clfA*	5-ACCCAGGTTCAGATTCTGGCAGCG-3	Z18852.1	60	165
	5-TCGCTGAGTCGGAATCGCTTGCT-3			
*clfB*	5-AACTCCAGGGCCGCCGGTTG-3	AJ224764.1	60	159
	5-CCTGAGTCGCTGTCTGAGCCTGAG-3			
*fib*	5-CGTCAACAGCAGATGCGAGCG-3	X72014.1	60	239
	5-TGCATCAGTTTTCGCTGCTGGTTT-3			
*ebps*	5-GGTGCAGCTGGTGCAATGGGTGT-3	U48826.2	60	191
	5-GCTGCGCCTCCAGCCAAACCT-3			
*eno*	5-TGCCGTAGGTGACGAAGGTGGTT-3	AF065394.1	60	195
	5-GCACCGTGTTCGCCTTCGAACT-3			
*cna*	5-AATAGAGGCGCCACGACCGT-3	M81736.1	60	156
	5-GTGCCTTCCCAAACCTTTTGAGCA-3			
*16S rRNA*	5-GGGACCCGCACAAGCGGTGG-3	L37597.1	60	191
	5-GGGTTGCGCTCGTTGCGGGA-3			

*fnbA* and *B*: fibronectin binding proteins A and B, *clfA* and *B*: clumping factors A and B, *fib*: fibrinogen binding protein, *eno*: laminin binding protein, *cna*: collagen binding protein, *ebps*: elastin binding protein, *icaADBC:* intercellular adhesion biofilm required genes, *16S rRNA* (housekeeping gene), * Optimized PCR annealing temperature program for all the genes at 60 °C.

**Table 3:** Program parameters used in the Protean IEF Cell.

Step	Voltage	Time	Voltage-Hours	Ramp
1	300	30 min	-	Liner
2	4000	2 h	-	Liner
3	4000	-	10,000	Rapid
Total	-	~5 h	~1400	-
Hold	500	3 h	-	Rapid

**Table 4:** MIC and 0.5 MIC of daptomycin and tigecycline for the individual *S. aureus* isolates used in this study.

Strain/Ho.Des.No	Antibiotic Concentration (µg/mL)			
	Tigecycline		Daptomycin	
	MIC	1/2 MIC ^a^	MIC	1/2 MIC ^a^
MRSA-527	0.25	0.125	0.5	0.25
MRSA-13	0.25	0.125	1	0.5
MRSA-139	0.5	0.25	1	0.5
MSSA-22d	0.125	0.06	0.125	0.06
MSSA-10E	0.125	0.06	1	0.5
MSSA-12E	0.125	0.06	2	1

^a^ Antibiotic concentrations added to individual *S. aureus* isolates used in this study.

**Table 5:** Relative expression of adhesion and biofilm target genes in cultures of six different isolates of *S. aureus* grown in the presence of sub- MIC daptomycinand sub-MIC tigecycline.

Gene	Type	0.5 MIC/daptomycin						0.5 MIC /Tigecycline					
		MSSA			MRSA			MSSA			MRSA		
		10E	12E	22d	527	13	139	10E	12E	22d	527	13	139
*16s*	REF	1.0	1.0	1.0	1.0	1.0	1.0	1.0	1.0	1.0	1.0	1.0	1.0
*fnb*A	TRG	1.8↑	1.0 *	0.0↓	1.5↑	1.5↑	0.4↓	1.1 *	3.4↑	0.1↓	1.6↑	1.8↑	0.4↓
*fnb*B	TRG	1.7↑	2.7↑	0.8 *	2.5↑	1.0 *	0.2↓	1.5↑	2.7↑	2.1↑	2.8↑	1.4↑	0.2↓
*clf*A	TRG	1.4 *	1.2↑	0.5↓	1.0 *	3.3↑	2.1↑	1.2 *	2.5↑	0.5↓	1.52↑	3.9↑	2.1↑
*clf*B	TRG	1.9↑	1.0 *	0.3↓	1.2 *	1.5↑	0.2↓	3.5↑	6.4↑	0.4↓	1.1 *	2.5↑	0.2↓
*Fib*	TRG	0.7↓	1.1 *	0.0↓	2.3↑	22.9↑	0.4↓	0.86 *	1.2 *	0.0↓	2.3↑	21.8↑	0.5↓
*eno*	TRG	1.0 *	7.9↑	0.6 *	1.8↑	22.9↑	0.2↓	1.05 *	9.7↑	0.5↓	2.3↑	21.9↑	0.3↓
*cna*	TRG	2.9↑	0.5↓	8.9↑	1.9↑	7.8↑	0.5↓	2.7↑	0.4↓	6.5↑	2.6↑	6.6↑	0.6↓
*ebp*s	TRG	0.9 *	2.9↑	0.1↓	1.9↑	10.9↑	0.4↓	1.2 *	3.1↑	0.1↓	1.5↑	7.7↑	0.4↓
*ica*A	TRG	0.8 *	1.1 *	0.0↓	4.8↑	1.1 *	0.4↓	0.6↓	1.0 *	0.0↓	5.6↑	7.0↑	0.4↓
*ica*D	TRG	1.5↑	1.9↑	0.0↓	1.2 *	2.6↑	0.3↓	1.3 *	1.9↑	0.0↓	1.2 *	3.9↑	0.3↓
*ica*B	TRG	1.6↑	0.9 *	0.0↓	1.3 *	2.7↑	0.6 *	1.3 *	0.8 *	0.0↓	1.4↑	3.4↑	0.6↓
*ica*C	TRG	2.5↑	0.9 *	0.0↓	1.4↑	2.1↑	0.2↓	1.9↑	0.5↓	0.0↓	2.0↑	2.7↑	0.2↓

Note: REF: reference gene, TRG: target gene, significant up regulation (↑) vs. significant down regulation (↓) (*p* < 0.05 unless indicated) of gene expression in the target sample compared to the control, * indicates that the expression between control and target sample was not significantly different (*p* > 0.05).

**Table 6:** Identification of highly significant down-regulated extracellular proteins in *S. aureus* clinical isolates MRSA (13), MRSA (527) and MRSA (139) after treatment with tigecycline by LC-MS.

Spot No.	Protein Name	Accession No.	Molecular Mass pI/Mw	Sequence Recovery
1	Putative uncharacterized protein	H1SYF7	5.29/21915.83	11%
2	Alkaline shock protein 23	H0DPE7	4.92/18648.79	16%
3	Alkyl hydroperoxide reductase subunit C	Q6GJR7	4.88/20976.61	25%
4	protein SA21194_0967	H0C9Z5	4.80/19312.74	12%
5	Superoxide dismutase	I0JDL1	5.08/22723.42	11%
6	Arabinose efflux permease family protein	H5XR59	8.94/46114.44	2%
7	Alcohol dehydrogenase, propanol-preferring	H1SYV0	5.24/35948.42	6%
8	Exotoxin 15	H4A246	8.45/26320.81	17%
9	Putative Cytochrome c4	D6CKS8	9.08/25250.81	7%
10	Putative septation protein spoVG	F0D890	4.79/10861.31	16%

## Supplementary Materials

The following are available online at https://www.mdpi.com/2079-6382/10/1/39/s1, Supplementary Section S1A: The specificity of each primer set for the amplification of adhesion and biofilm genes in this study, Supplementary Section S1B: Part of the raw cDNA sequence files of the targeted biofilm genes (*icaA,D,B,C*), adhesion genes (*clfA*, *clfB*, *fnbA*, *fnbB*, *fib*, *ebps*, *eno*, *cna*), and *16sRNA* housekeeping region from MRSA-clone 527 sequenced directly from RT-PCR products, Supplementary Section S2A1: Relative Expression Ratio of 12 target genes in MSSA (10E), exposed to sub-MIC of daptomycin, Supplementary Section S2A2: Relative Expression Ratio of 12 target genes in MSSA (12E), exposed to sub-MIC of daptomycin, Supplementary Section S2A3; Relative Expression Ratio of 12 target genes in MSSA (22-d), exposed to sub-MIC of daptomycin, Supplementary Section S2A4: Relative Expression Ratio of 12 target genes in MRSA (139), exposed to sub-MIC of daptomycin, Supplementary Section S2A5: Relative Expression Ratio of 12 target genes in MRSA (13), exposed to sub-MIC of daptomycin, Supplementary Section S2A6: Relative Expression Ratio of 12 target genes in MRSA (527), exposed to sub-MIC of daptomycin, Supplementary Section S2B1: Relative Expression Ratio of 12 target genes in MSSA (10E), exposed to sub-MIC of tigecycline, Supplementary Section S2B2: Relative Expression Ratio of 12 target genes in MSSA (12E), exposed to sub-MIC of tigecycline, Supplementary Section S2B3: Relative Expression Ratio of 12 target genes in MSSA (22d), exposed to sub-MIC of tigecycline, Supplementary Section S2B4: Relative Expression Ratio of 12 target genes in MRSA (139), exposed to sub-MIC of tigecycline, Supplementary Section S2B5: Relative Expression Ratio of 12 target genes in MRSA (13), exposed to sub-MIC of tigecycline, Supplementary Section S2B6: Relative Expression Ratio of 12 target genes in MRSA (527), exposed to sub-MIC of tigecycline, Supplementary Section S3A: Comparing 2D gel protein patterns of MSSA clinical isolates with and without tigecycline treatment, Supplementary Section S3B: Comparing 2D gel protein patterns of MRSA clinical isolates with and without tigecycline treatment.
