# Supplementary material for: The Role of Subinhibitory Concentrations of Daptomycin and Tigecycline in Modulating Virulence in Staphylococcus aureus
Source: Antibiotics (Basel). 2021 Jan 3;10(1):39. doi: 10.3390/antibiotics10010039 (PMC7823975; doi:10.3390/antibiotics10010039)

antibiotics

MDPI

Supplementary Materials
The Role of Subinhibitory Concentrations of Daptomycin and Tigecycline in Modulating Virulence in *Staphylococcus aureus*

Salman Sahab Atshan [1,2,3,4,*], Rukman Awang Hamat [2,*], Marco J. L. Coolen [5], Gary Dykes [3], Zamberi Sekawi [2], Benjamin J. Mullins [3], Leslie Thian Lung Than [2], Salwa A. Abduljaleel [6] and Anthony Kicic [3,4,7,8]

Supplementary S1

A.The specificity of each primer set for the amplification of adhesion and biofilm genes in this study. (**A**). *Melting curve analyses* were used at the end of q PCR products amplifications to confirm that there was only one peak and only one product. (**B**). End amplified products of real-time (RT-PCR) were visualized after separation on an agarose gel, showing all primer pairs resulted in amplification of a single product of *ica*A(1), *ica*D(2), *ica*B(3), *ica*C(4), *fnb*A(5), *fnb*B(6), *clf*A(7), *clf*B(8), *fib*(9), *ebpS*(10), *eno*(11), *cna*(12), and *16s*(13), M, molecular weight marker.
B.Part of the raw cDNA sequence files of the targeted biofilm genes (*icaA,D,B,C*),adhesion genes (*clfA, clfB, fnbA, fnbB,fib, ebps, eno, cna*), and *16sRNA* housekeeping region from MRSA-clone 527 sequenced directly from RT-PCR products.

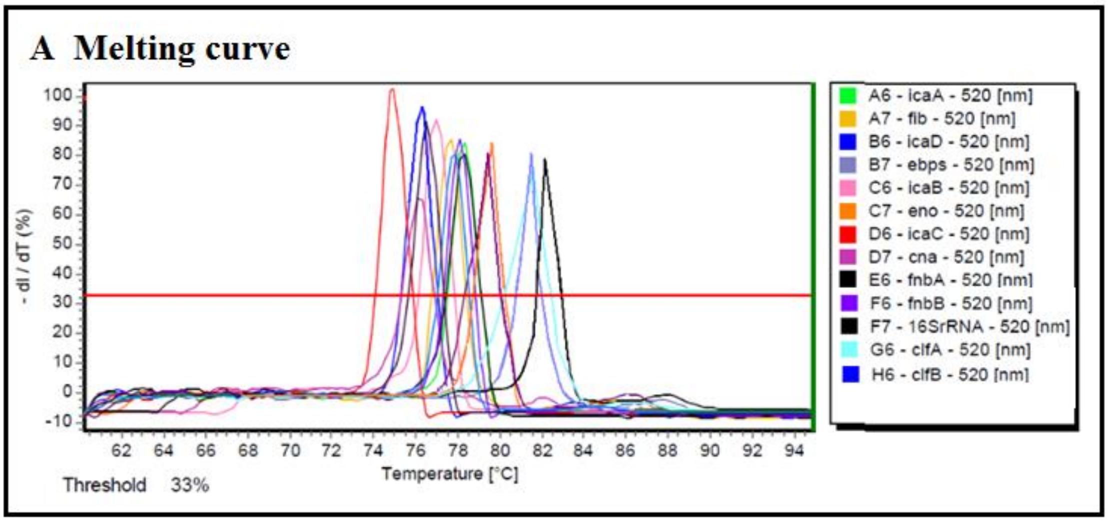

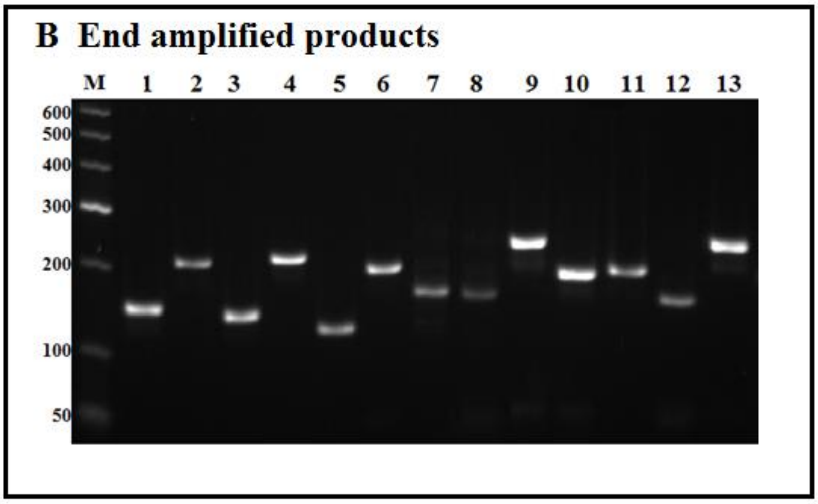

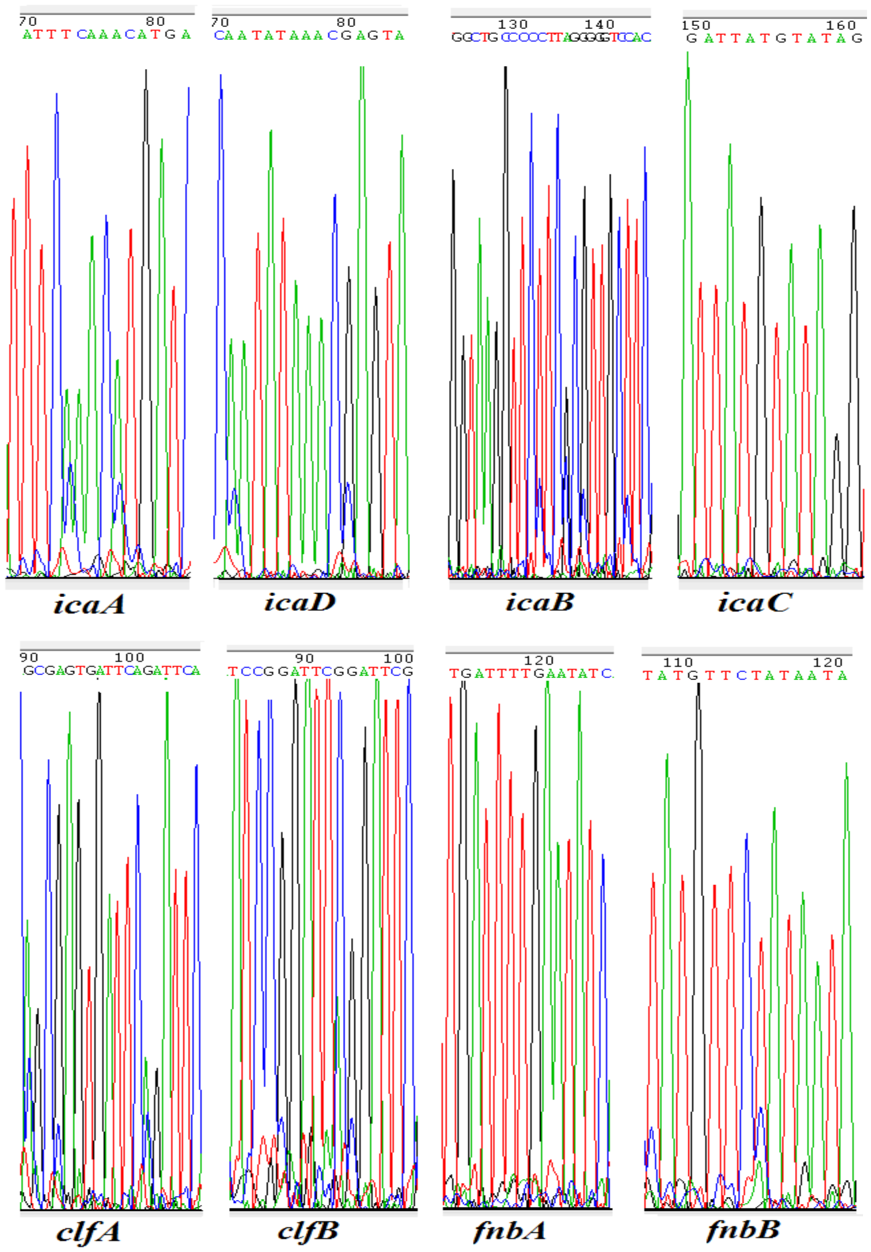
icaA
icaD
icaB
icaC
clfA
clfB
fnbA
fnbB

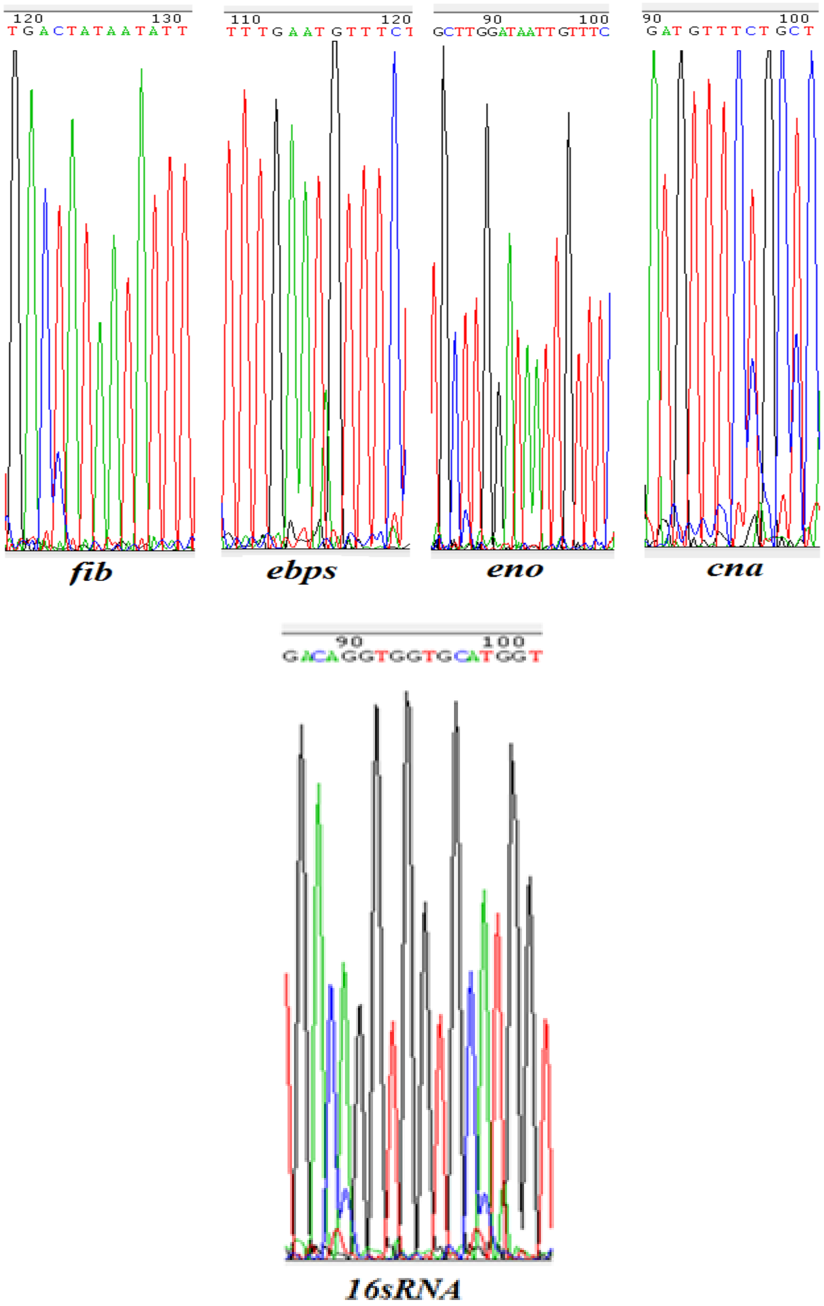

fib
ebps
eno
cna
16sRNA

Supplementary S2

A1. Relative Expression Ratio of 12 target genes in MSSA (10E), exposed to sub-MIC of daptomycin.

Gene	Type	Expression	P value
16s	REF	1.000	
*fnb*A	TRG	1.895	**0.000**
*fnb*B	TRG	1.752	**0.000**
*clf*A	TRG	1.455	0.191
*clf*B	TRG	1.955	**0.001**
Fib	TRG	0.766	**0.002**
eno	TRG	1.048	0.657
cna	TRG	2.955	**0.001**
*ebp*s	TRG	0.967	0.743
*ica*A	TRG	0.864	0.060
*ica*D	TRG	1.575	**0.001**
*ica*B	TRG	1.649	**0.040**
*ica*C	TRG	2.575	**0.000**

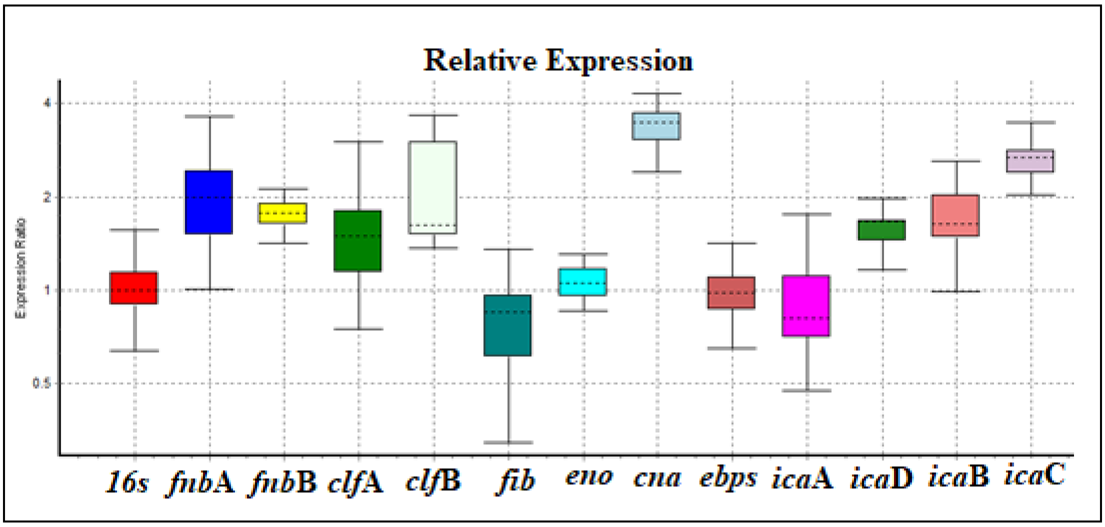

NOTE: REF: reference gene, TRG: target gene, bold numerical values indicated significantly up or down regulated genes, significance if P value is <0.05.

A2. Relative Expression Ratio of 12 target genes in MSSA (12E), exposed to sub-MIC of daptomycin.

Gene	Type	Expression	P value
16s	REF	1.000	
*fnb*A	TRG	1.085	0.633
*fnb*B	TRG	2.795	**0.000**
*clf*A	TRG	1.233	**0.022**
*clf*B	TRG	1.072	0.373
Fib	TRG	1.178	0.359
eno	TRG	7.99	**0.001**
cna	TRG	0.512	0.001
ebps	TRG	2.954	**0.001**
*ica*A	TRG	1.154	0.441
*ica*D	TRG	1.996	**0.003**
*ica*B	TRG	0.965	0.849
*ica*C	TRG	0.912	0.384

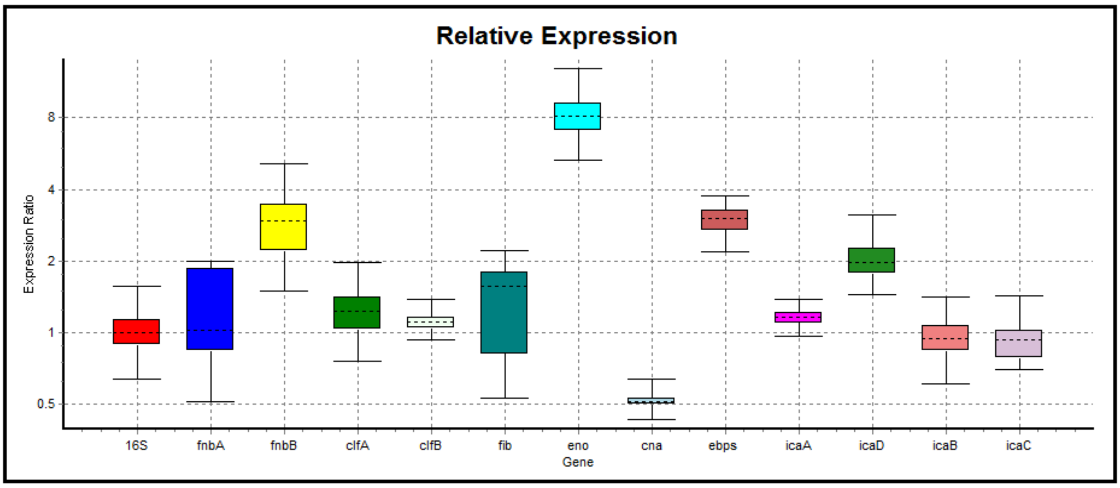

NOTE: REF: indicates reference gene, TRG: target gene, bold numerical values indicated significantly up or down regulated genes, significance UP to significantly increased, and DOWN to significantly decreased if P value is <0.05.

A3. Relative Expression Ratio of 12 target genes in MSSA (22-d), exposed to sub-MIC of daptomycin.

Gene	Type	Expression	P value
16s	REF	1.000	
*fnb*A	TRG	0.074	**0.000**
*fnb*B	TRG	0.892	0.849
*clf*A	TRG	0.587	**0.000**
*clf*B	TRG	0.322	**0.001**
Fib	TRG	0.065	**0.000**
eno	TRG	0.688	0.055
cna	TRG	8.996	**0.001**
ebps	TRG	0.127	**0.000**
*ica*A	TRG	0.058	**0.000**
*ica*D	TRG	0.022	**0.000**
*ica*B	TRG	0.046	**0.000**
*ica*C	TRG	0.039	**0.001**

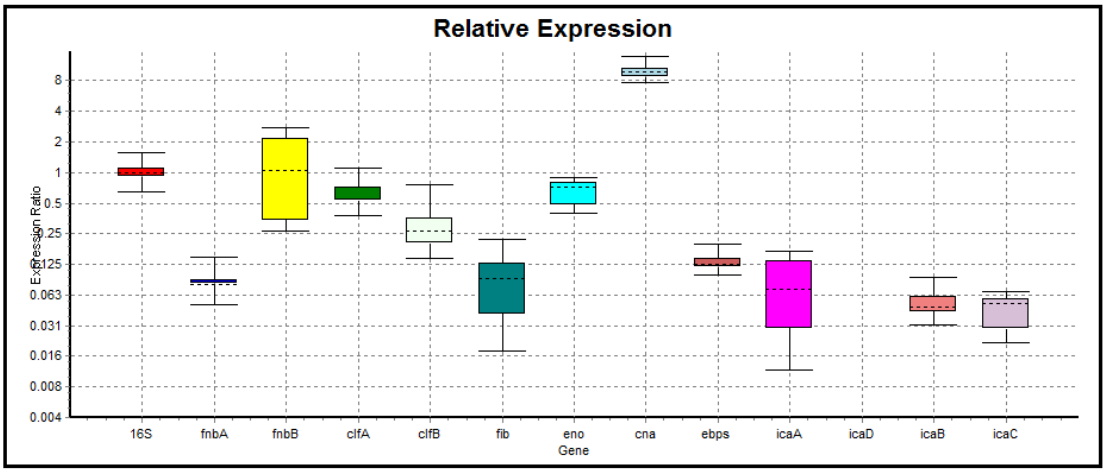

NOTE: REF: indicates reference gene, TRG: target gene, bold numerical values indicated significantly up or down regulated genes, significance UP to significantly increased, and DOWN to significantly decreased if P value is <0.05.

A4. Relative Expression Ratio of 12 target genes in MRSA (139), exposed to sub-MIC of daptomycin.

Gene	Type	Expression	P value
16s	REF	1.000	
*fnb*A	TRG	0.400	**0.000**
*fnb*B	TRG	0.233	**0.001**
*clf*A	TRG	2.187	**0.001**
*clf*B	TRG	0.290	**0.000**
Fib	TRG	0.442	**0.002**
eno	TRG	0.256	**0.001**
cna	TRG	0.549	**0.046**
*ebp*s	TRG	0.423	**0.000**
*ica*A	TRG	0.415	**0.000**
*ica*D	TRG	0.399	**0.000**
*ica*B	TRG	0.635	0.078
*ica*C	TRG	0.277	**0.001**

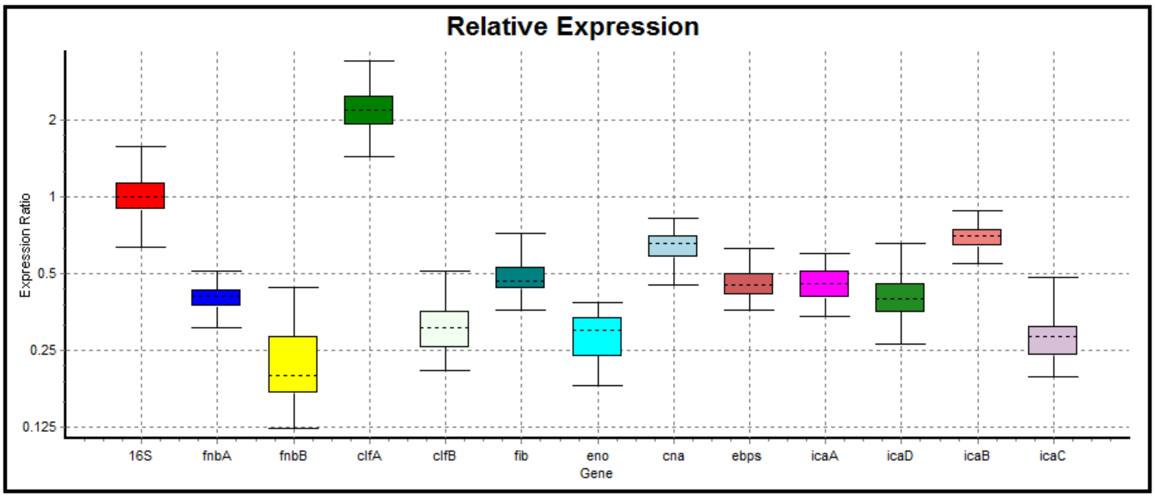

NOTE: REF: reference gene, TRG: target gene, bold numerical values indicated significantly up or down regulated genes, significance if P value is <0.05.

A5. Relative Expression Ratio of 12 target genes in MRSA (13), exposed to sub-MICof daptomycin.

Gene	Type	Expression	P value
16s	REF	1.000	
*fnb*A	TRG	1.550	**0.048**
*fnb*B	TRG	1.059	**0.752**
*clf*A	TRG	3.30	**0.002**
*clf*B	TRG	1.55	**0.015**
Fib	TRG	22.98	**0.001**
eno	TRG	22.94	**0.001**
cna	TRG	7.89	**0.001**
*ebp*s	TRG	10.90	**0.001**
*ica*A	TRG	1.108	0.731
*ica*D	TRG	2.698	**0.001**
*ica*B	TRG	2.785	**0.000**
*ica*C	TRG	2.166	**0.001**

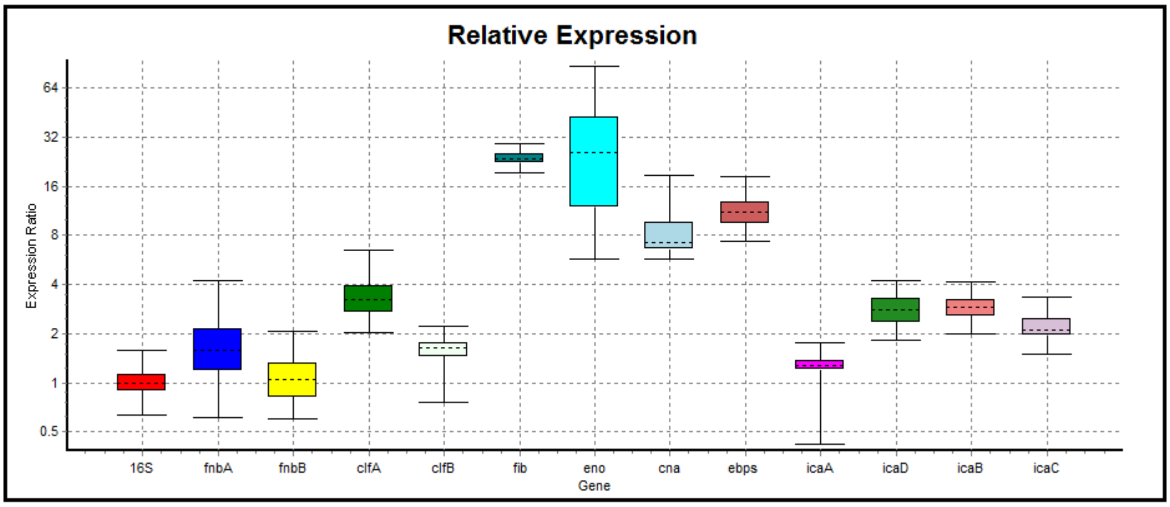

NOTE: REF: reference gene, TRG: target gene, bold numerical values indicated significantly up or down regulated genes, significance if P value is <0.05.

A6. Relative Expression Ratio of 12 target genes in MRSA (527), exposed to sub-MIC ofdaptomycin.

Gene	Type	Expression	P value
16s	REF	1.000	
*fnb*A	TRG	1.523	**0.007**
*fnb*B	TRG	2.522	**0.001**
*clf*A	TRG	1.059	0.611
*clf*B	TRG	1.236	0.296
Fib	TRG	2.38	**0.000**
eno	TRG	1.83	**0.000**
cna	TRG	1.96	**0.037**
*ebp*s	TRG	1.90	**0.039**
*ica*A	TRG	4.88	**0.000**
*ica*D	TRG	1.22	0.098
*ica*B	TRG	1.32	0.011
*ica*C	TRG	1.45	**0.146**

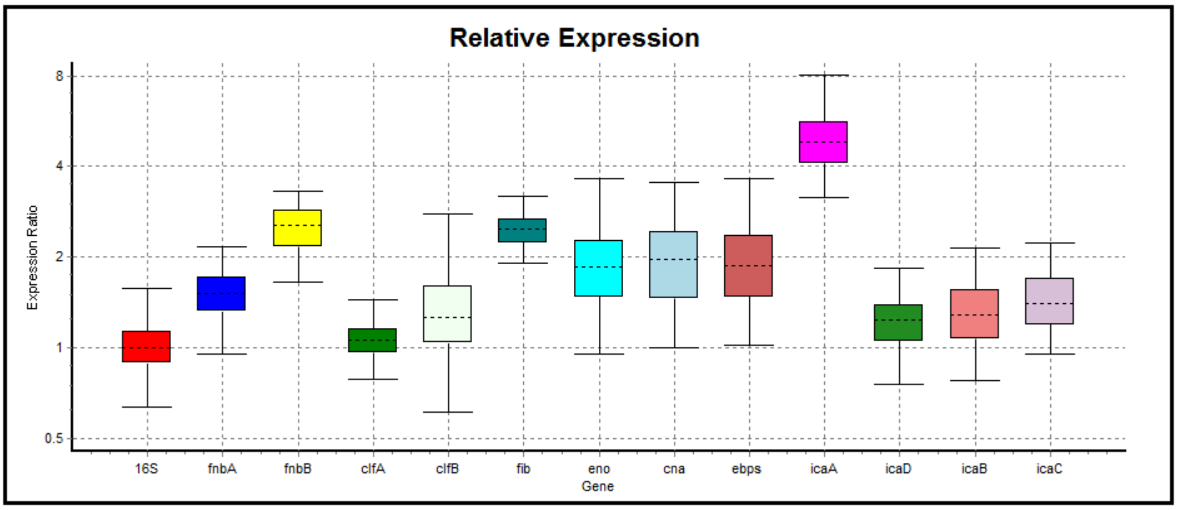

NOTE: REF: reference gene, TRG: target gene, bold numerical values indicated significantly up or down regulated genes, significance if P value is <0.05.

B1. Relative Expression Ratio of 12 target genes in MSSA (10E), exposed to sub-MIC of Tigcycline.

Gene	Type	Expression	P value
16s	REF	1.000	
fnbA	TRG	1.158	0.559
fnbB	TRG	1.556	**0.002**
clfA	TRG	1.246	0.274
clfB	TRG	3.510	**0.001**
Fib	TRG	0.861	0.667
eno	TRG	1.058	0.528
cna	TRG	2.770	**0.001**
ebps	TRG	0.897	0.559
icaA	TRG	0.595	**0.017**
icaD	TRG	1.344	0.143
icaB	TRG	1.359	0.115
icaC	TRG	1.908	**0.035**

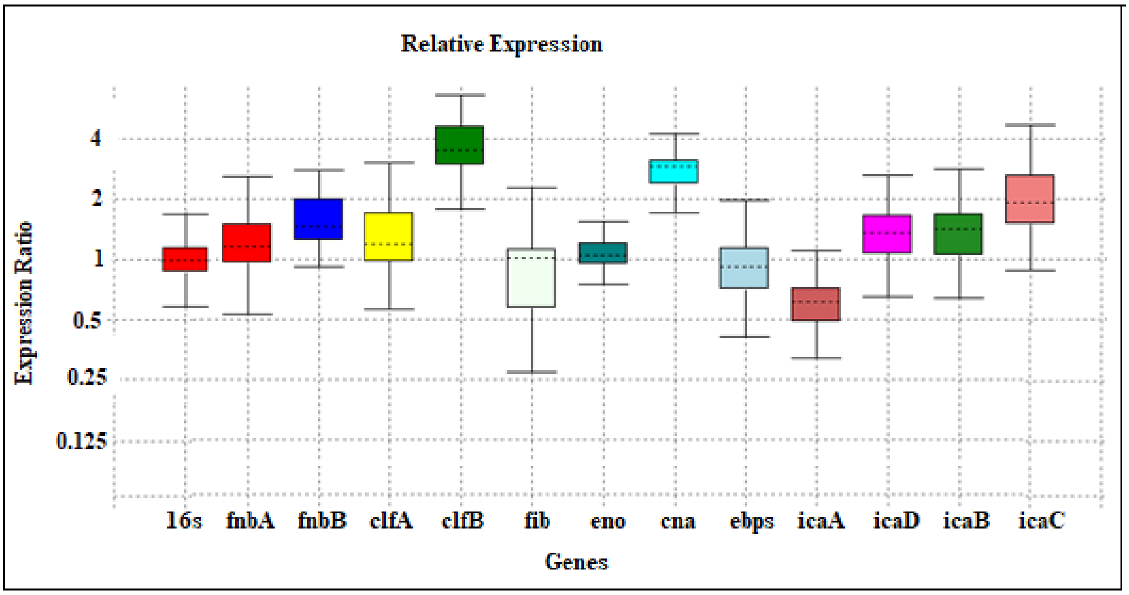

NOTE: REF: reference gene, TRG: target gene, bold numerical values indicated significantly up or down regulated genes, significance if P value is <0.05.

B2. Relative Expression Ratio of 12 target genes in MSSA (12E), exposed to sub-MIC of tigecycline.

Gene	Type	Expression	P value
16s	REF	1.000	
fnbA	TRG	3.438	**0.000**
fnbB	TRG	2.785	**0.001**
clfA	TRG	2.504	**0.001**
clfB	TRG	6.422	**0.001**
Fib	TRG	1.280	0.116
eno	TRG	9.701	**0.001**
cna	TRG	0.459	0.000
ebps	TRG	3.158	**0.001**
icaA	TRG	1.097	0.499
icaD	TRG	1.929	**0.001**
icaB	TRG	0.813	0.219
icaC	TRG	0.598	**0.039**

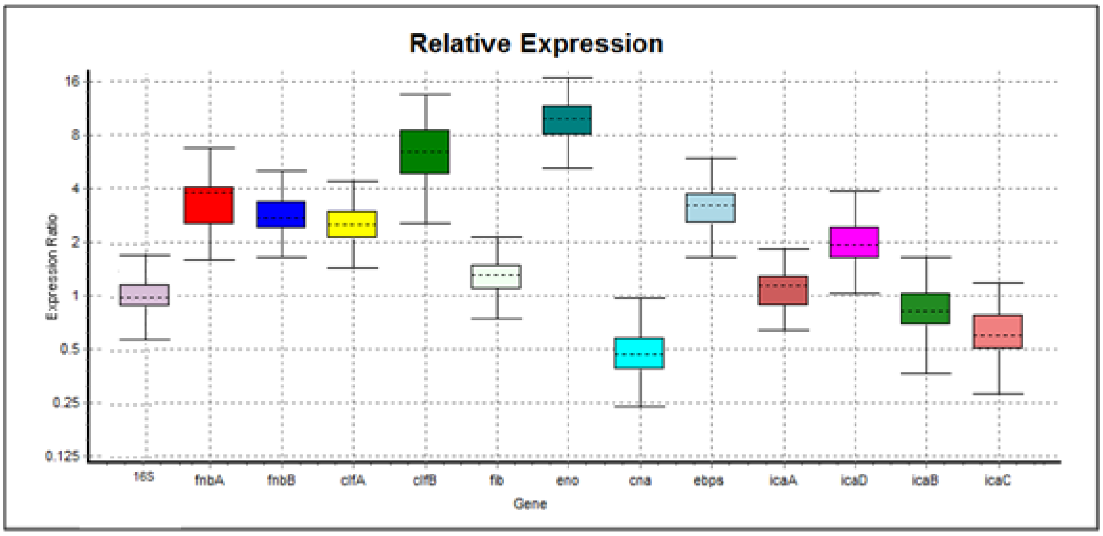

NOTE: REF: reference gene, TRG: target gene, bold numerical values indicated significantly up or down regulated genes, significance if P value is <0.05.

B3. Relative Expression Ratio of 12 target genes in MSSA (22d), exposed to sub-MIC of tigecycline.

Gene	Type	Expression	P value
16s	REF	1.000	
fnbA	TRG	0.109	**0.001**
fnbB	TRG	2.128	**0.001**
clfA	TRG	0.563	**0.006**
clfB	TRG	0.455	**0.001**
Fib	TRG	0.027	**0.000**
eno	TRG	0.575	**0.039**
cna	TRG	6.578	**0.001**
ebps	TRG	0.136	**0.000**
icaA	TRG	0.063	**0.001**
icaD	TRG	0.023	**0.001**
icaB	TRG	0.054	**0.001**
icaC	TRG	0.021	**0.001**

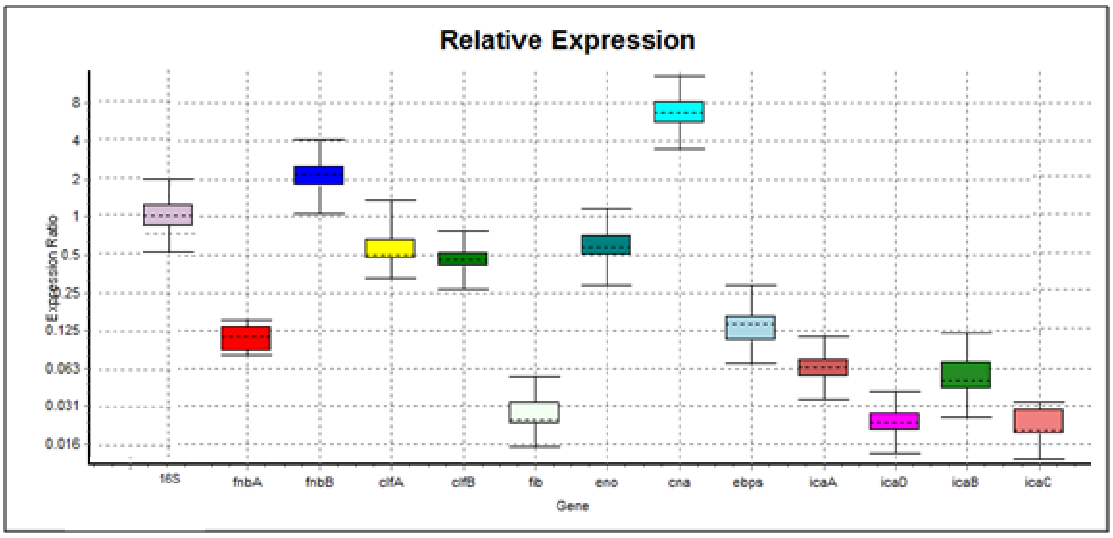

NOTE: REF: reference gene, TRG: target gene, bold numerical values indicated significantly up or down regulated genes, significance if P value is <0.05.

B4. Relative Expression Ratio of 12 target genes in MRSA (139), exposed to sub-MIC of tigecycline.

Gene	Type	Expression	P value
16s	REF	1.000	
fnbA	TRG	0.404	**0.001**
fnbB	TRG	0.205	**0.001**
clfA	TRG	2.120	**0.006**
clfB	TRG	0.287	**0.002**
Fib	TRG	0.550	**0.012**
eno	TRG	0.324	**0.001**
cna	TRG	0.691	**0.039**
ebps	TRG	0.489	**0.001**
icaA	TRG	0.448	**0.002**
icaD	TRG	0.369	**0.001**
icaB	TRG	0.670	**0.004**
icaC	TRG	0.266	**0.001**

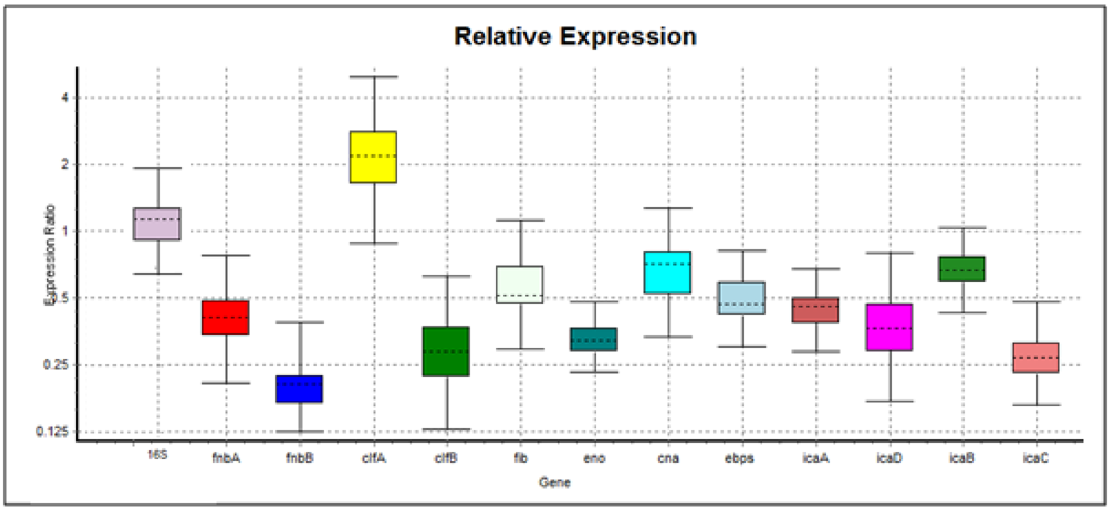

NOTE: REF: reference gene, TRG: target gene, bold numerical values indicated significantly up or down regulated genes, significance if P value is <0.05.

B5. Relative Expression Ratio of 12 target genes in MRSA (13), exposed to sub-MIC of tigecycline.

Gene	Type	Expression	P value
16s	REF	1.000	
fnbA	TRG	1.832	**0.005**
fnbB	TRG	1.498	**0.038**
clfA	TRG	3.945	**0.002**
clfB	TRG	2.549	**0.000**
Fib	TRG	21.849	**0.001**
eno	TRG	21.981	**0.002**
cna	TRG	6.616	**0.001**
ebps	TRG	7.743	**0.000**
icaA	TRG	7.010	**0.001**
icaD	TRG	3.931	**0.002**
icaB	TRG	3.456	**0.002**
icaC	TRG	2.716	**0.001**

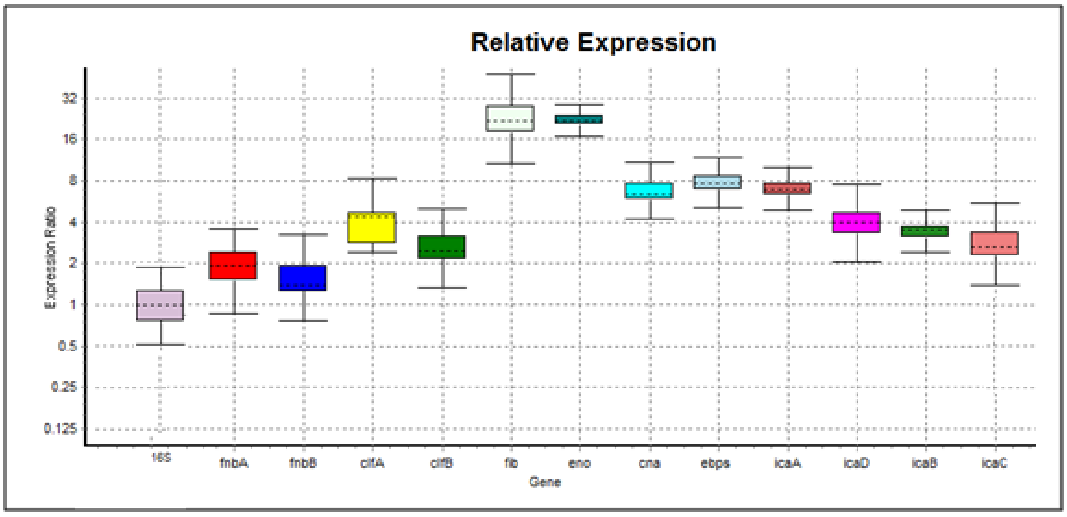

NOTE: REF: reference gene, TRG: target gene, bold numerical values indicated significantly up or down regulated genes, significance if P value is <0.05.

B6. Relative Expression Ratio of 12 target genes in MRSA (527), exposed to sub-MIC of tigecycline.

Gene	Type	Expression	P value
16s	REF	1.000	
fnbA	TRG	1.662	**0.022**
fnbB	TRG	2.870	**0.001**
clfA	TRG	1.522	**0.009**
clfB	TRG	1.186	0.151
Fib	TRG	2.368	**0.001**
eno	TRG	2.372	**0.002**
cna	TRG	2.646	**0.001**
ebps	TRG	1.564	**0.038**
icaA	TRG	5.606	**0.000**
icaD	TRG	1.293	0.091
icaB	TRG	1.452	**0.012**
icaC	TRG	2.038	**0.000**

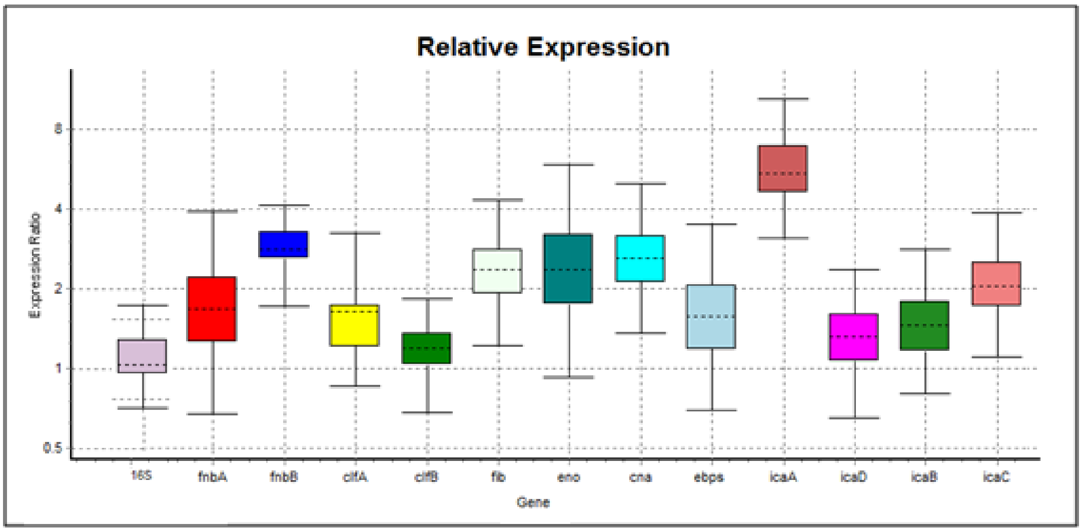

NOTE: REF: reference gene, TRG: target gene, bold numerical values indicated significantly up or down regulated genes, significance if P value is <0.05.

Supplementary S3

A.Comparing 2D gel protein patterns of MSSA clinical isolates with and without tigecycline treatment. In total 25µg of the protein extract of each isolate was separated on 2D gels, using IPG strips (pI 4–7). Protein spots were stained with silver stain and scanned using Densitometer GS-800 Mode Imager.

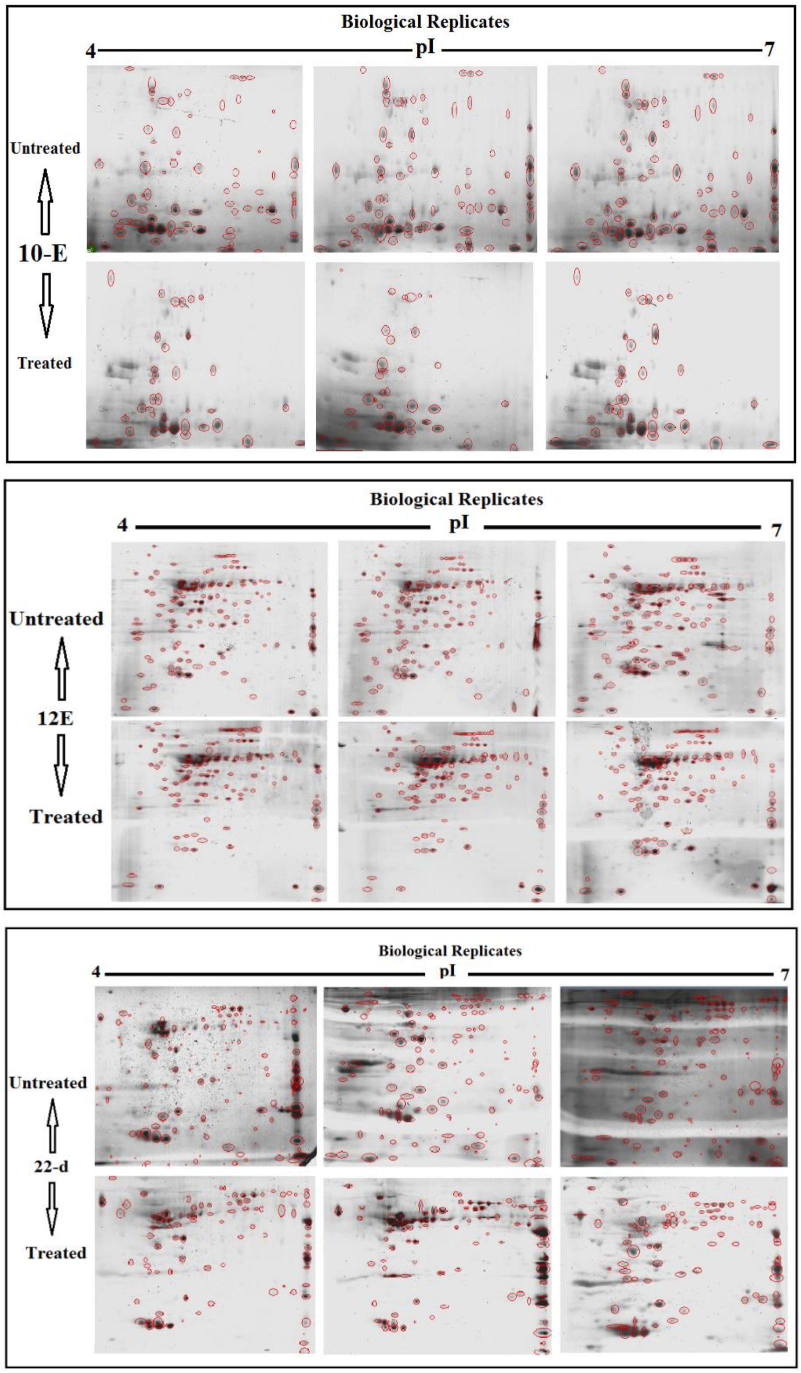

B.Comparing 2D gel protein patterns of MRSA clinical isolates with and without tigecycline treatment. In total 25µg of the protein extract of each isolate was separated on 2D gels, using IPG strips (pI 4–7). Protein spots were stained with silver stain and scanned using Densitometer GS-800 Mode Imager.

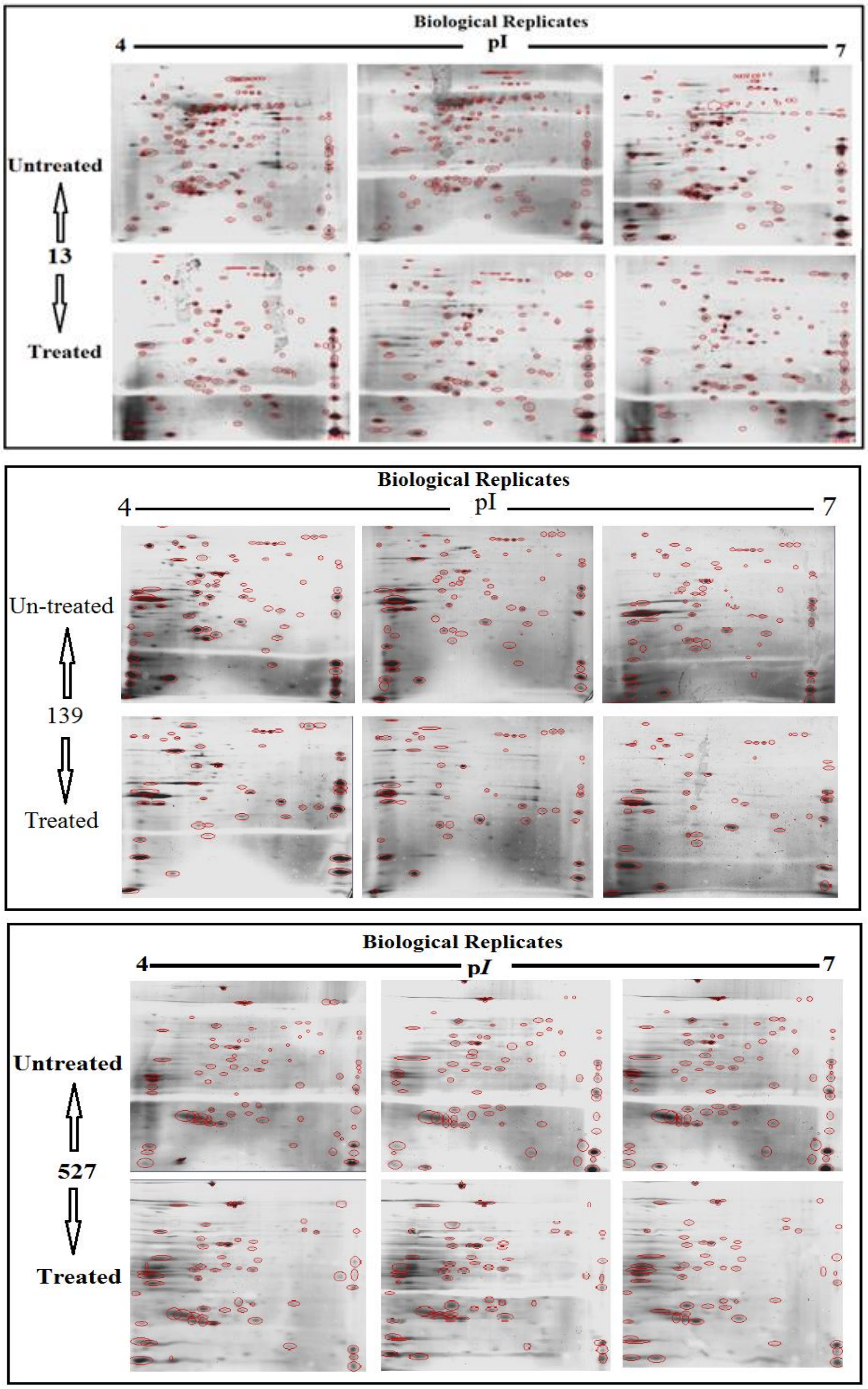

Supplement: Supplementary file 1 [file antibiotics-10-00039-s001.pdf]
